# Using Convolutional Neural Network with Taguchi Parametric Optimization for Knee Segmentation from X-Ray Images

**DOI:** 10.1155/2021/5521009

**Published:** 2021-08-23

**Authors:** Young Jae Kim, Seung Ro Lee, Ja-Young Choi, Kwang Gi Kim

**Affiliations:** ^1^Department of Biomedical Engineering, Gil Medical Center, Gachon University College of Medicine, Incheon 21565, Republic of Korea; ^2^Department of Radiology, Seoul National University Hospital, Seoul 03080, Republic of Korea

## Abstract

Loss of knee cartilage can cause intense pain at the knee epiphysis and this is one of the most common diseases worldwide. To diagnose this condition, the distance between the femur and tibia is calculated based on X-ray images. Accurate segmentation of the femur and tibia is required to assist in the calculation process. Several studies have investigated the use of automatic knee segmentation to assist in the calculation process, but the results are of limited value owing to the complexity of the knee. To address this problem, this study exploits deep learning for robust segmentation not affected by the environment. In addition, the Taguchi method is applied to optimize the deep learning results. Deep learning architecture, optimizer, and learning rate are considered for the Taguchi table to check the impact and interaction of the results. When the Dilated-Resnet architecture is used with the Adam optimizer and a learning rate of 0.001, dice coefficients of 0.964 and 0.942 are obtained for the femur and tibia for knee segmentation. The implemented procedure and the results of this investigation may be beneficial to help in determining the correct margins for the femur and tibia and can be the basis for developing an automatic diagnosis algorithm for orthopedic diseases.

## 1. Introduction

Knee osteoarthritis (OA) is a disease in which knee cartilage is lost for various reasons including aging, knee injury, and obesity such that the bones of the femur and tibia are in contact with each other [1, 2]. The global prevalence of knee osteoarthritis was 22.9% in individuals aged 40 and over. Accordingly, there are around 654.1 million individuals (40 years and older) with knee osteoarthritis in 2020 worldwide [3]. Given that knee epiphysis is a chronic disease that cannot be completely cured, early diagnosis and frequent checks of cartilage status are important [4]. Radiographic X-ray images are normally used in the diagnosis process because the shadows of bones are readily identified [5, 6].

The calculation of Joint Space Width (JSW) is a useful method for evaluating the progression of arthritis using X-ray images [7]. JSW facilitates the calculation of the gap width between the femur and tibia of the knee. The exact margins of the femur and tibia must be defined to measure accurate JSW. Especially, in the development of JSW's automatic measurement algorithm, accurate segmentation of the femur and tibia is important. However, a separate approach is required to accurately segment the femur and tibia in X-ray images. Previous studies on automatic knee segmentation have shown that the segmentation process is not accurate due to the complexity of the shape of the knee [8] and the inside of the joint cannot be accurately segmented along the outer line of the knee [9]. Therefore, it is necessary to develop an algorithm that can robustly segment the region regardless of the complexity of the knee.

Deep learning technology has been widely applied to a variety of fields using advanced hardware and software technologies. Among them, convolutional neural network (CNN) is a technology that is mainly used in the field of imaging. CNN recognizes the patterns extracted using a filter and accumulates them repeatedly, so that even complex patterns can be recognized [10, 11]. Given that robust results are obtained irrespective of the image, the limitations of existing image processing techniques can be addressed. Therefore, studies based on the application of CNN to medical imaging using the previously described features are continuously being reported.

In 2018, Novikov et al. attempted to segment images of the lungs using the U-Net model. The original U-Net, All-Dropout U-Net, All-Convolutional U-Net, and InvertedNet architecture are used for deep learning with 247 X-ray images in the JSRT database. All Drop U-Net model shows the highest dice coefficient of 0.988 [12]. In 2018, Kolařík et al. used Dense U-Net, Res-U-Net, and U-Net models for brain image segmentation. In total, 252 brain images were used for deep learning. Comparing the results, Dense U-Net yielded the highest dice coefficient of 0.988 [13]. In 2016, Ben-Cohen et al. used FCN-8s DAG net and FCN-4s DAG net for image segmentation. Based on learning and verifying using the 20 Liver CT scan datasets of the SLIVER07 challenge, the FCN-8s DAG net with adjacent slices yielded the highest dice coefficient of 0.870 for the CT image segmentation [14].

These studies show that using deep learning yields superior performance compared to previous image processing techniques. Research has also been conducted on the effect of changing various parameters on performance. However, there is a tendency to change only one parameter, such as the model architecture. There are few reports on the simultaneous consideration of several parameters such as the model, optimizer, and learning rate, and there are no reports on the segmentation of X-ray images of the knee. In this study, we attempt to identify the optimized combination of parameters for knee segmentation of X-ray images by applying the Taguchi method. The main objective is to derive the best results to accurately segment these images.

## 2. Materials and Methods

### 2.1. Development Environment

In this report, Microsoft Visual Studio (Ver. 2010, Microsoft, Redmond, WA, USA) was used for image preprocessing of the data. The deep learning system consisted of two NVIDIA GeForce GTX 1080Ti (NVIDIA, Santa Clara, Calif) GPU, Xeon E5-1650 v4 (Intel, Santa Clara, Calif) CPU, and 64 GB RAM. Deep learning was performed using the Ubuntu 14.04 operating system via the Python 2.7.6 and Keras 2.1.5 (with TensorFlow backend) framework.

### 2.2. Data

This study was approved by the institutional review board of the Seoul National University Hospital (SNUH), and the requirement for informed consent was waived for this retrospective study. For enrolment of study subjects, a musculoskeletal radiologist (J.Y.C. with 16 years of experience in knee imaging) initially searched our picture archiving and communication system (PACS) for patients who took the standing anteroposterior radiograph of both knees in SNUH between January and March 2018. Excluding the cases with fractures, surgery, or inflammatory or infectious arthritis, 285 knee standing anteroposterior radiographs including 570 knees were finally selected. The severity of the knee OA according to the Kellgren-Lawrence X-ray grading system was as follows: grade 0 (normal), 131 knees; grade 2 (doubtful OA), 154 knees; grade 2 (mild OA), 178 knees; grade 3 (moderate OA), 81 knees; and grade 4 (severe OA), 22 knees. Among these, 35 knee radiographs including 70 knees (grade 0, 14 knees; grade 1, 52 knees; and grade 2, 2 knees) were used as a test set. All knee radiographs were divided into 570 each knee sections. The DICOM standard was used in the modified images with pixel values of 16 bits and a size of 1,172 pixels in the horizontal direction and 2,118 pixels in the vertical direction.

Deep learning via CNN requires that the input images should have the same width and height. Therefore, in this investigation, the images were resized to 256 pixels by 256 pixels during preprocessing. Augmentation was performed to increase the amount of data. The condition for Augmentation was set to 5% for both the up/down and the left/right movement ratio in addition the enlargement/reduction range of the original image. By performing this process 10 times on the original images using a maximum rotation angle of 5° and the left and right reversal conditions, a total of 5,300 images of the femur and tibia were obtained.

To create the training data, an expert directly demarked the region of interest (ROI) of the femur and radius in the knee image. The directly drawn ROI was the mask image and that was used as the correct data for learning and verification. The femur, which is the object of the region of interest, is an upper bone with rounded features, while the tibia has a flat, upper-bony feature. In addition, the fibula where is next to the tibia is sometimes marked on the X-ray image of the knee.

Of the 5300 sections of data, 5,000 sections were used for deep learning, with 4,000 sections of learning data and 1,000 sections used for verifying. The remaining 300 images were used for final verification of the deep learning model.

### 2.3. Convolutional Neural Network

**Figure 1:**
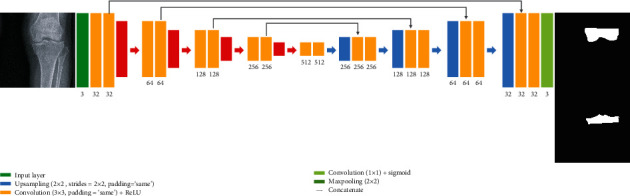
U-Net architecture for knee segmentation.

**Figure 2:**
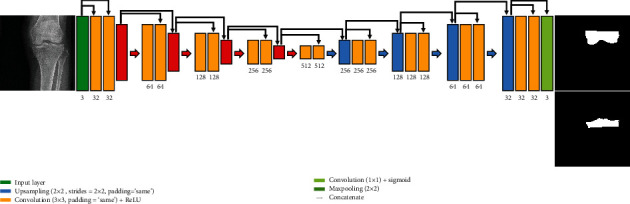
Dense U-Net architecture for knee segmentation.

**Figure 3:**
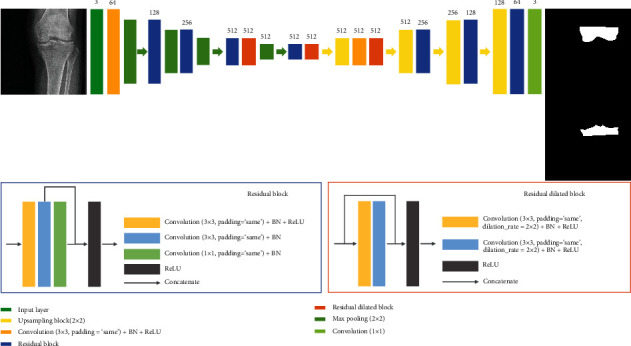
Dilated-Resnet architecture for knee segmentation.

In this study, the models used for segmentation are U-Net, Dilated-Resnet, and Dense U-Net. U-Net and Dense U-Net are shown in Figures 1 and 2. Dense U-Net applies to concatenate to all feature maps that pass through the convolution layer in the U-Net model. Dilated-Resnet is shown in Figure 3.

U-Net is a modified model based on a Fully Connected Network that enables more accurate detection even with a small number of training images [15]. This model has a U-shaped network structure and consists of a contracting path to gradually reduce the image and an expanding path to expand the image. U-Net can speed up the verification process because it skips previously verified components and verifies the next patch component instead of using a sliding window, which verifies components for a second time. However, given that the contracting path does not involve a padding process, the outer region of the image is gradually cut off. This problem can be solved via the mirror padding method because the image to be cut has a symmetric structure with respect to the boundary axis. Image compensation processing is performed by cropping the data of the contracting path to an appropriate size followed by concatenation (moving) to save the path lost when mirror padding is performed. Based on such a series of processes, more precise detection can proceed at a faster rate.

Dilated-Resnet is a model that is created by adding a dilated convolution block to the Resnet structure. Information for various scales can be obtained without difficulty because dilated convolution increases the size of the receptive field. In addition, only the points at specific positions are used for the operations, and the remaining positions are filled with zeros. As such, the number of operations can be decreased [16]. The convolution layer used in the contracting path of this study consists of 3 × 3 consecutive convolution layers and a 1 × 1 convolution layer for the padding process. The dilated convolution block applies a 3 × 3 convolution layer and a dilation rate of (2, 2) to obtain a 7 × 7 receptive field.

Finally, Dense U-Net achieves a high density by applying concatenation to all feature maps that pass through the convolution layer [17]. The structure is the same as the previous U-Net; however, the feature maps of the previous stage are accumulated continuously in the U-Net structure. It is therefore expected that information in the entire network can be efficiently acquired. In the final layer, three channels were output as a 1 × 1 convolution.

### 2.4. Design of Experiments

The Taguchi method was used in this investigation, which minimizes the number of experiments and identifies the main variables that affect the results [18, 19]. The parameters used in the experiment are shown in Table 1 as the architecture, optimizer, and learning rate.

Three levels are set for each parameter. The levels used in the architecture are U-Net, Dilated-Resnet, and Dense U-Net, as proven models. As the optimizer to minimize the loss function, Momentum with an emphasis on direction [20], Adadelta with speed [21], and Adam which emphasizes speed and direction [22] were used. The commonly used value of 0.9 was set for Momentum in this study. The learning rates have three levels with values of 0.01, 0.001, and 0.0001.

Given that it takes time for the optimizer to find the weight according to the learning rate and the progress of the architecture is also affected, it is assumed that there is an interaction between the architecture and the learning rate and the optimizer and the learning rate. The calculated degree of freedom (DOF) is 3 × (3 − 1) + 2 × (3 − 1) × (3 − 1) = 14. Therefore, we used the Taguchi table L_18 with a DOF of 17 [23].  Table 2 shows the experimental table used in this investigation.

## 3. Results

In this investigation, we experimented the femur and tibia segmentation for x-ray images of the knee using a composite neural network with parameters according to the Taguchi technique as shown in Table 2. To determine the performance of the learned model, we utilized verification data with the learning model and confirmed the results.  Table 3 shows the dice coefficients for the femur and tibia divisions based on the experiment. The highest dice coefficients were determined to be 0.964 and 0.942 in both the femur and tibia, respectively, when architecture is the Dilated-Resnet architecture, optimizer is the Adam, and learning rate is the value of 0.001 which is Run-14 condition.

Tables 4 and 5 are the response tables of the parameters of the femur and tibia. The tables show that parameter B and the interaction between parameter A, architecture, and parameter C, and the learning rate have the greatest effect on the results as indicated by the high delta values for both the femur and tibia.

When using Adam as an optimizer, the highest dice coefficient was 0.953 for the femur and 0.926 for the tibia on average.  Table 6 shows the interaction matrix between the architecture and the learning rate in the femur and tibia identified in Tables 4 and 5. In both cases, when the architecture is Dilated-Resnet and the learning rate is 0.001, the highest dice coefficients are recorded as 0.960 and 0.935, respectively.

**Figure 4:**
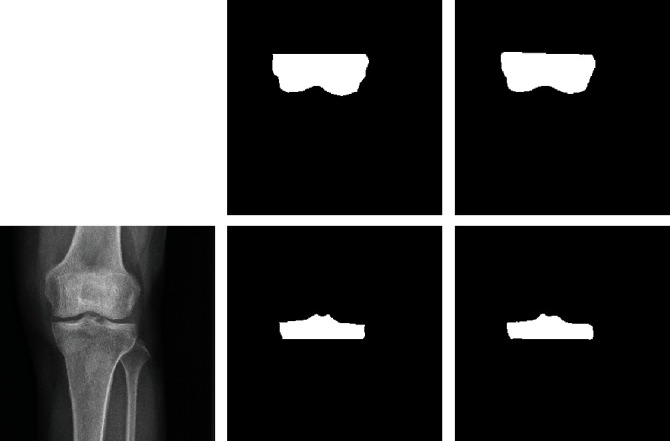
Comparison of knee segmentation between manual and deep learning model. (a) Original. (b) Manual segmentation results. (c) Deep learning model segmentation results.

The response table and interaction matrix show that the optimized condition is the Run-14 condition which performs best in Table 3.  Figure 4 shows the manual segmentation results by experts and the deep learning results by the Run-14 conditions.

**Figure 5:**
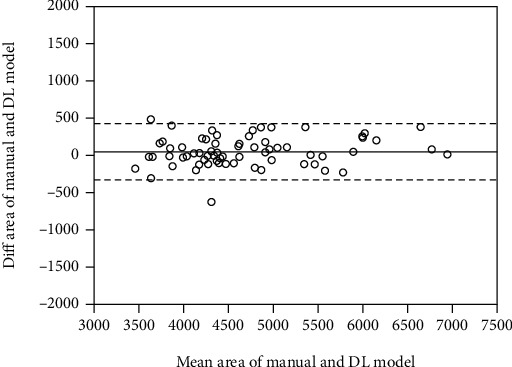
Bland-Altman plot for femur.

**Figure 6:**
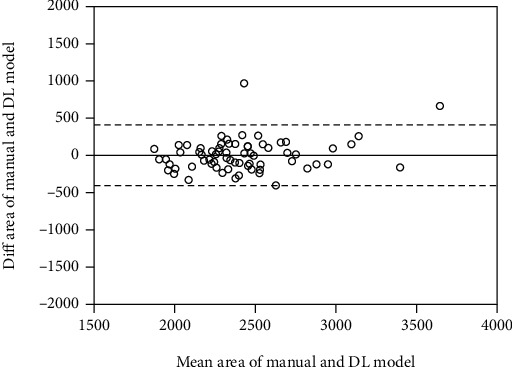
Bland-Altman plot for tibia.

Figures 5 and 6 are the Bland-Altman plots obtained by calculating the areas for the manual segmentation results and the deep learning results. In the Bland-Altman plot analysis, equivalence is achieved because most values are within ±1.96 standard deviations from the mean for each area difference.

## 4. Discussion

In this investigation, the experiments were designed using the Taguchi method for X-ray images of the knee, and the performance of the model was verified by determining the optimized conditions that yielded the best segmentation results using a convolutional neural network. In the experimental results obtained for the Taguchi method, when the architecture is Dilated-Resnet, the optimizer is Adam, and the learning rate is 0.001, the highest dice coefficient was obtained. By analyzing the response table and interaction matrix, the experimental results were verified and the aforementioned conditions resulted in the best results.

In addition, Bland-Altman plot analysis was performed by comparing the results of the best performance condition of Dilated-Resnet, Adam, and 0.001 to the results of manual segmentation. It was determined that most of the values were within the standard deviation and it was thus established that the deep learning model can be useful.

As a result of deep learning, the result for the tibia was lower than that for the Femur. This is due to the flat and complex structure of the tibia and the presence of the fibula. It was possible to obtain good results using only 530 images, but this might be insufficient data for deep learning. Therefore, it is necessary to collect additional images of various patients.

Both femur and tibia, Run-9 and Run-15 conditions were unable to segment the knee. In Run-3 and Run-12 which have the same conditions for the architecture and optimizer, the dice coefficient was 0.951 for the femur and 0.923 for the tibia. Therefore, it is considered that the learning rate applied to the Run-9 and Run-15 conditions is low for knee segmentation. Run-16 exhibited lower dice coefficients of 0.245 and 0.106 for the femur and tibia, respectively. It was also determined to have a low learning rate. This is because the case of Run-4 with the same architecture and optimizer, but a different learning rate yielded the dice coefficients of 0.738 and 0.610, respectively. Using the Taguchi method, low-performing conditions were determined to be effective and it is better to avoid Run-9, 15, and 16. If additional variables such as the loss function are added in the future, it is thought that a more optimized division result could be obtained under various conditions.

In general, when considering various architectures and parameters in deep learning research, it is necessary to experiment with all possible combinations, and experimenting with all combinations requires a lot of time and effort. On the other hand, using the Taguchi method, it is possible to efficiently derive the best combination of architectures and parameters while reducing unnecessary experiments.

In conclusion, the correlation between architecture, optimizer, and learning rate was considered in this study. These are the main variables of deep learning in knee segmentation. For the three conditions in both the femur and tibia, 54 times of deep learning is required. However, 36 times are required in the case of the Taguchi method. As such, the best-optimized conditions could be derived. If additional studies are performed, statistical analysis using variance analysis or the Taguchi method by adding other parameters can be used to obtain more accurate results. The method proposed in this paper can be helpful in determining the correct margins for the femur and tibia and can be the basis for developing an automatic diagnosis algorithm for orthopedic disease.

## Figures and Tables

**Table 1:** Parameters for experiments.

Parameters	Levels		
	1	2	3
(a) Architecture	U-Net	Dilated-Resnet	Dense U-Net
(b) Optimizer	Adam	Adadelta	Momentum
(c) Learning rate	0.01	0.001	0.0001

**Table 2:** Experimental table used for the Taguchi method.

Run	A	A∗C	B	B∗C	C
1	1	1	1	1	1
2	2	2	2	2	1
3	3	3	3	3	1
4	1	1	2	2	2
5	2	2	3	3	2
6	3	3	1	1	2
7	1	2	1	3	3
8	2	3	2	1	3
9	3	1	3	2	3
10	1	3	3	2	1
11	2	1	1	3	1
12	3	2	2	1	1
13	1	2	3	1	2
14	2	3	1	2	2
15	3	1	2	3	2
16	1	3	2	3	3
17	2	1	3	1	3
18	3	2	1	2	3

**Table 3:** Results of experiments.

Run	1	2	3	4	5	6	7	8	9
DSC_Femur	0.939	0.950	0.951	0.738	0.956	0.937	0.956	0.696	0.000
DSC_Tibia	0.918	0.925	0.929	0.609	0.927	0.896	0.938	0.401	0.000
Run	10	11	12	13	14	15	16	17	18
DSC_Femur	0.892	0.962	0.951	0.940	0.964	0.000	0.245	0.945	0.961
DSC_Tibia	0.822	0.927	0.923	0.903	0.942	0.000	0.106	0.926	0.936

**Table 4:** Response table for femur.

Femur	A	B	C	A∗C	B∗C
Level1	0.785	0.953	0.941	0.597	0.901
Level2	0.912	0.597	0.756	0.952	0.751
Level3	0.633	0.781	0.634	0.781	0.679
Delta	0.279	0.357	0.307	0.355	0.223

**Table 5:** Response table for tibia.

Tibia	A	B	C	A∗C	B∗C
Level1	0.716	0.926	0.908	0.564	0.828
Level2	0.841	0.494	0.713	0.925	0.706
Level3	0.614	0.751	0.551	0.683	0.638
Delta	0.227	0.432	0.356	0.362	0.190

**Table 6:** Interaction matrix between architecture and learning rate.

Femur	A1	A2	A3	Tibia	A1	A2	A3
C1	0.915	0.956	0.951	C1	0.870	0.926	0.926
C2	0.839	0.960	0.469	C2	0.756	0.935	0.448
C3	0.601	0.821	0.480	C3	0.522	0.663	0.468
Femur	A1	A2	A3	Tibia	A1	A2	A3

## Data Availability

The datasets analyzed during this study are available from the corresponding author on request.
